# Validation of the DIGIROP-birth model in a Chinese cohort

**DOI:** 10.1186/s12886-021-01952-0

**Published:** 2021-05-27

**Authors:** Sizhe Chen, Rong Wu, He Chen, Wenbei Ma, Shaolin Du, Chao Li, Xiaohe Lu, Songfu Feng

**Affiliations:** 1 Department of Ophthalmology, Zhujiang Hospital, Southern Medical University, No.253 Gongyedadao Middle Road, Guangzhou, 510282 Guangdong China; 2 Department of Ophthalmology, Peking Union Medical College Hospital, Beijing, China; 3 Department of Ophthalmology, Tung Wah Hospital, Sun Yat-sen University, Dongguan, China

**Keywords:** Retinopathy of prematurity, Prediction, Retina

## Abstract

**Background:**

We aimed to validate the predictive performance of the DIGIROP-Birth model for identifying treatment-requiring retinopathy of prematurity (TR-ROP) in Chinese preterm infants to evaluate its generalizability across countries and races.

**Methods:**

We retrospectively reviewed the medical records of preterm infants who were screened for retinopathy of prematurity (ROP) in a single Chinese hospital between June 2015 and August 2020. The predictive performance of the model for TR-ROP was assessed through the construction of a receiver-operating characteristic (ROC) curve and calculating the areas under the ROC curve (AUC), sensitivity, specificity, and positive and negative predictive values.

**Results:**

Four hundred and forty-two infants (mean (SD) gestational age = 28.8 (1.3) weeks; mean (SD) birth weight = 1237.0 (236.9) g; 64.7% males) were included in the study. Analyses showed that the DIGIROP-Birth model demonstrated less satisfactory performance than previously reported in identifying infants with TR-ROP, with an area under the receiver-operating characteristic curve of 0.634 (95% confidence interval = 0.564–0.705). With a cutoff value of 0.0084, the DIGIROP-Birth model showed a sensitivity of 48/93 (51.6%), which increased to 89/93 (95.7%) after modification with the addition of postnatal risk factors. In infants with a gestational age < 28 weeks or birth weight < 1000 g, the DIGIROP-Birth model exhibited sensitivities of 36/39 (92.3%) and 20/23 (87.0%), respectively.

**Conclusions:**

Although the predictive performance was less satisfactory in China than in developed countries, modification of the DIGIROP-Birth model with postnatal risk factors shows promise in improving its efficacy for TR-ROP. The model may also be effective in infants with a younger gestational age or with an extremely low birth weight.

**Supplementary Information:**

The online version contains supplementary material available at 10.1186/s12886-021-01952-0.

## Background

Retinopathy of prematurity (ROP) is a retinal vasoproliferative disease that affects preterm infants. Although it is avoidable through early diagnosis and timely treatment, it is a leading cause of childhood blindness [1]. The risk of blindness from ROP is as high as 40% in developing countries, while it is less than 10% in developed countries [2]. Insufficient neonatal and ophthalmologic care, as well as variation in clinical practice, may account for the varying neonatal outcomes associated with ROP [3]. Further, noncompliance with follow-up examinations is one of the major causes of blindness from ROP, particularly in developing countries [4]. Under the current screening criteria, less than 10% of screened infants require treatment for ROP [5]. Effective implementation of ROP screening is hindered by limited medical resources in developing countries or regions and over-screening under varying recommended guidelines [1–3, 5]. Thus, there is a pressing need for prediction models for ROP that safely ease the workload of screening.

Low gestational age and birth weight are the major risk factors for ROP, and form the basis of most established screening guidelines [1]. Prediction models have also been developed that incorporate additional clinical parameters, such as weight gain rate, race, and respiratory distress syndrome [6–9]. However, according to a 2016 report by the American Academy of Ophthalmology, none of these prediction models are clinically applicable due to limited generalizability and small sample size [10]. Although some models have been validated and exhibited excellent predictive performance, the validation cohorts consisted mainly of infants in highly developed countries [11–19]. Nevertheless, the data gathered from less-developed countries are scarce but indispensable. These data indicate that racial variation exists, with Asian infants being at greater risk of ROP than White infants, suggesting a genetic predisposition to ROP in addition to the underlying socioeconomic factors associated with low birth weight, small for gestational age, and preterm birth [20, 21]. For example, the WINROP model, based on weekly weight gain [6], has been widely validated around the globe. It displayed favorable utility in predicting severe ROP in developed countries [14–16], but performed less satisfyingly in moderately or less-developed countries such as China, Mexico, and Turkey [17–19]. Furthermore, the implementation of higher oxygen saturation targets (i.e., 91–95%) in neonatal intensive care units may reduce the impact of poor weight gain as a risk factor for ROP and has been shown to diminish the WINROP model’s predictive ability [22]. Thus, ROP risk prediction models should be validated across different races and countries or regions with various clinical settings.

Recently, Pivodic et al. proposed an individualized risk prediction model for treatment-requiring retinopathy of prematurity (TR-ROP), called DIGIROP-Birth. The model was based on gestational age, birth weight, and sex and applicable for infants with a gestational age of 24 to 30 weeks [23]. It has several strengths that suggest its potential for clinical application [24]. First, the model was accessible online as a risk calculator, without any input of postnatal factors. This easy-to-use model enabled early identification of high-risk infants and early planning of ROP examination and follow-up schedules, thereby improving compliance with ROP screening examinations and decreasing visual impairment from ROP. Second, the model was internally and externally validated in large multicenter cohorts consisting of infants born in developed countries and exhibited promising predictive performance. However, no validation has been reported in low- or middle- income countries or in race-specific cohorts.

Therefore, the present study aimed to validate the DIGIROP-Birth model in Chinese preterm infants to evaluate its generalizability across countries and races.

## Methods

### Study design

Preterm infants who received ROP screening examination between June 2015 and August 2020 in the neonatal intensive care unit of Zhujiang Hospital of Southern Medical University, Guangzhou, China, were retrospectively recruited. The study was approved by the hospital’s institutional ethics committee and adhered to the tenets of the Declaration of Helsinki. All parents or guardians of the recruited infants provided written informed consent prior to participation. Data were anonymized and de-identified before analysis. According to the Chinese guideline for ROP screening, eligible participants were preterm infants with gestational ages less than 32 weeks, birth weight less than 2000 g, or with risk factors for ROP as determined by a neonatologist [25]. Infants with incomplete data or any other ocular diseases besides ROP were excluded. Also, infants with gestational ages less than 24 weeks or beyond 30 weeks were further excluded as the DIGIROP-Birth model was not developed for application in these gestational ages. The diagnosis of ROP and indication of treatment for ROP followed the International Classification of ROP Revisited and the Early Treatment for ROP Study, respectively [26, 27].

Clinical data collected for our study related to maternal factors, neonatal factors, and neonatal interventions. These data were extracted from medical records by SZ and R. Specifically, maternal factors of interest included maternal age, mode of delivery (cesarean or vaginal), multiple gestations, in vitro fertilization, gestational hypertension, gestational diabetes mellitus, reproductive tract infection during pregnancy, and use of antenatal steroids. Neonatal factors included gestational age, birth weight, sex, apnea, respiratory distress syndrome, bronchopulmonary dysplasia, sepsis, necrotizing enterocolitis, intraventricular hemorrhage, patent ductus arteriosus, anaemia, and hyperbilirubinemia. Finally, neonatal interventions included invasive mechanical ventilation and blood transfusions.

### Statistical analysis

Categorical variables were expressed as numbers and percentages and analyzed using chi-squared tests. Continuous variables were expressed as means and standard deviations and compared using Wilcoxon’s rank-sum tests. *P* < 0.05 (two-tailed) was considered statistically significant. To identify the independent risk factors for TR-ROP, univariate logistic regression analyses were performed for each variable. Variables with a *P* < 0.1 in univariate logistic analyses were included in multivariate logistic regression analyses. To assess the predictive performance of the model for TR-ROP, a receiver-operating characteristic (ROC) curve was constructed. In the ROC analysis, the areas under the ROC curve (AUC), sensitivity, specificity, positive predictive value (PPV), and negative predictive value (NPV) were calculated. The cutoff value that resulted in the maximal sum of sensitivity and specificity was chosen and assessed. In addition, the cutoff values previously tested by Pivodic et al. were also selected for validation [23]. The performance of the DIGIROP-Birth model for identifying TR-ROP infants was also evaluated by gestational age and birth weight. Specifically, the study cohort was divided into gestational age < 28 weeks (extremely preterm infant) and gestational age ≥ 28 weeks subgroups, and birth weight < 1000 g (extremely low birth weight) and birth weight ≥ 1000 g subgroups. Statistical analyses were performed using SPSS 20.0 (IBM Corp., Armonk, NY, USA).

## Results

Of the 732 infants who underwent ROP screening, 11 had incomplete data and five had ocular diseases besides ROP. Among the remaining 716 infants, 274 were further excluded due to a gestational age less than 24 weeks or beyond 30 weeks, resulting in 442 infants being included in the study. Two hundred thirty-seven of 442 participants (53.6%) developed ROP of any stage, of whom 93 of 442 (21.0%) required treatment. The mean (SD) gestational age at birth was 28.8 (1.3) weeks, the mean (SD) birth weight was 1237.0 (236.9) g, and the sex distribution was 286 (64.7%) male and 156 (35.3%) female. Clinical characteristics of the study population are summarized in Table 1. Compared with infants without TR-ROP, gestational age and birth weight were significantly lower (both *P* < 0.001) among TR-ROP cases. A significant difference in sex was also observed between the groups (*P* = 0.046), where the proportion of males was significantly higher in infants with TR-ROP. Finally, there was a higher occurrence of apnea (*P* = 0.041) and intraventricular hemorrhage (*P* = 0.034) in infants with TR-ROP.

**Table 1:** Clinical characteristics of the study cohort

Variables	No TR-ROP (*n* = 349)	TR-ROP (*n* = 93)	*P*
	Mean (SD)	Mean (SD)	
**Maternal factors**			
Maternal age, years			0.451
25–30	125 (36.9)	30 (33.0)	
< 25	60 (17.7)	16 (17.6)	
30–35	101 (29.8)	29 (31.9)	
> 35	53 (15.6)	16 (17.6)	
Caesarean delivery	135 (38.7)	29 (31.2)	0.183
Multiple gestations	81 (23.2)	15 (16.1)	0.141
In vitro fertilization	43 (12.3)	10 (10.8)	0.679
Gestational hypertension	23 (6.7)	10 (10.8)	0.175
Gestational diabetes	54 (15.5)	15 (16.1)	0.877
Reproductive tract infections during pregnancy	31 (8.9)	5 (5.4)	0.272
Antenatal steroids use	82 (23.5)	14 (15.1)	0.079
**Neonatal factors**			
Gestational age, weeks	29.0 ± 1.2	28.2 ± 1.5	< 0.001
Birth weight, g	1267.8 ± 230.5	1121.5 ± 226.3	< 0.001
Male	234 (67.0)	52 (55.9)	0.046
Apnea	97 (27.8)	36 (38.7)	0.041
Respiratory distress syndrome	263 (75.4)	75 (80.6)	0.285
Bronchopulmonary dysplasia	197 (56.4)	62 (66.7)	0.075
Sepsis	105 (30.1)	27 (29.0)	0.891
Necrotizing enterocolitis	36 (10.3)	14 (15.1)	0.2
Intraventricular hemorrhage	130 (37.2)	46 (49.5)	0.034
Patent ductus arteriosus	130 (37.2)	36 (38.7)	0.796
Anaemia	178 (51.0)	49 (52.7)	0.804
Hyperbilirubinemia	215 (61.6)	53 (57.0)	0.418
**Neonatal interventions**			
Invasive mechanical ventilation	325 (93.1)	87 (93.5)	0.885
Blood transfusions	305 (87.4)	88 (94.6)	0.074

*SD* Standard deviation, *TR-ROP* Treatment-requiring retinopathy of prematurity

Univariate and multivariate logistic regression analyses were conducted to identify risk factors for TR-ROP (Supplementary Table 1). Among the maternal factors, only antenatal steroid use was associated with TR-ROP (*P* = 0.082). Among the neonatal factors, infants with TR-ROP had a younger gestational age and lower birth weight (both *P* < 0.001) than infants without TR-ROP. In addition, male sex (*P* = 0.047), apnea (*P* = 0.043), bronchopulmonary dysplasia (*P* = 0.077) and intraventricular hemorrhage (*P* = 0.035) were associated with TR-ROP. As for the neonatal interventions, only blood transfusions were associated with TR-ROP (*P* = 0.082). Multivariate logistic regression analysis found that gestational age (odds ratio [OR] = 0.758; 95% confidence interval [CI] = 0.587–0.979; *P* = 0.034), birth weight (OR = 0.261; 95% CI = 0.107–0.635; *P* = 0.016), male sex (OR = 1.767; 95% CI = 1.058–2.952; *P* = 0.030), apnea (OR = 2.013; 95% CI = 1.305–3.156; *P* = 0.020), and intraventricular hemorrhage (OR = 3.617; 95% CI = 1.365–8.521; *P* = 0.009) were independently associated with TR-ROP.

**Fig. 1:**
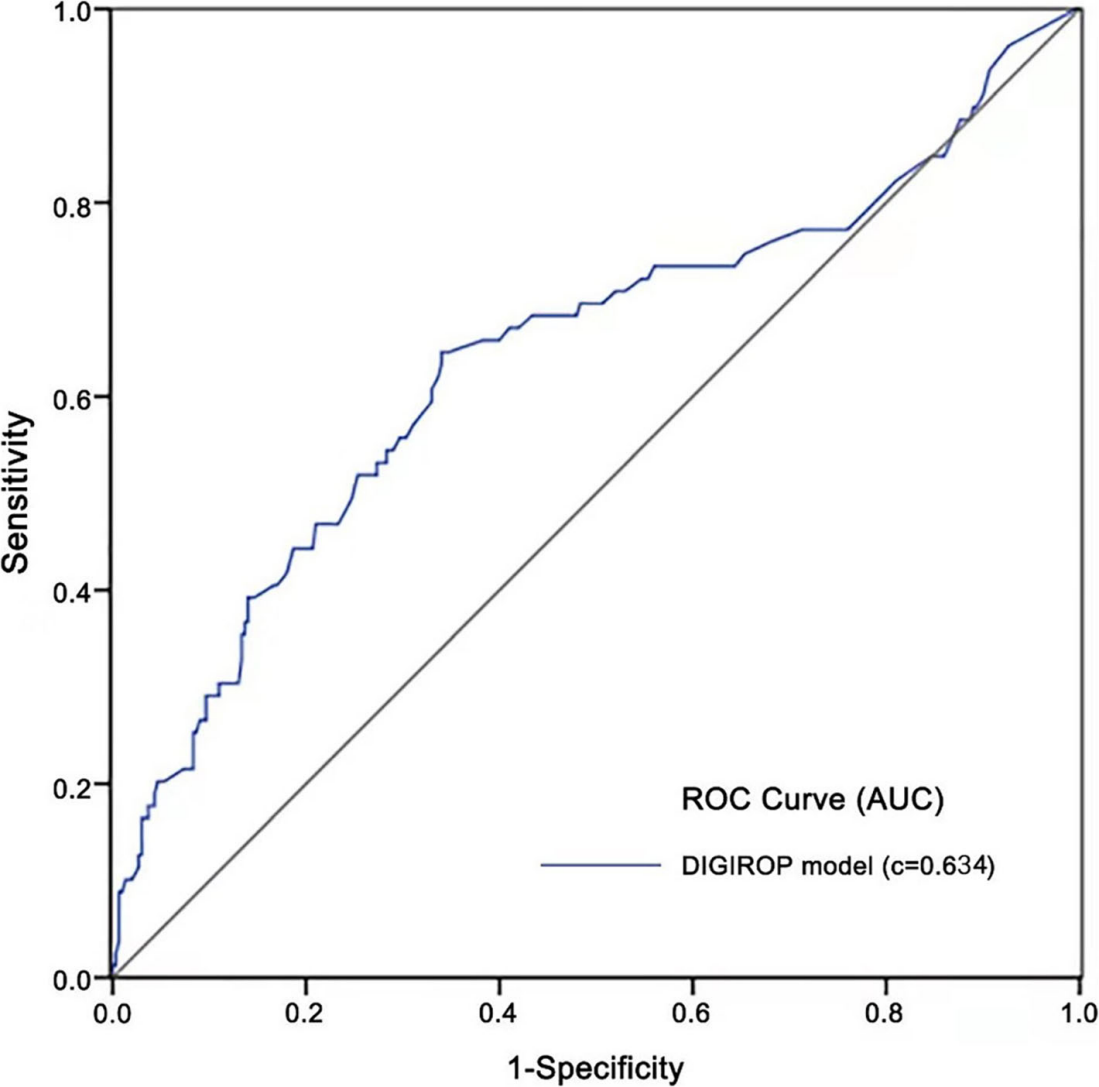
ROC curve of the DIGIROP-Birth model for TR-ROP prediction in the Chinese cohort. ROC, receiver-operating characteristic; TR-ROP, treatment-requiring retinopathy of prematurity; AUC, area under the receiver operating characteristic curve

The DIGIROP-Birth model demonstrated unsatisfactory performance for identifying infants with TR-ROP, with an AUC of 0.634 (95% CI, 0.564–0.705) (Fig. 1). The predictive performances of the DIGIROP-Birth model with different cutoff values are displayed in Table 2. When applying a cutoff value of 0.0084, which resulted in a maximal sum of sensitivity and specificity, the DIGIROP-Birth model demonstrated a sensitivity of 48/93 (51.6%) and an NPV of 263/308 (85.4%). Among those who were determined to be at risk of TR-ROP below 0.84% by the DIGIROP-Birth model, 45 of 299 (15.1%) infants developed ROP that required treatment. Forty-one of these 45 (91.1%) infants had at least one premature birth complication, including apnea and intraventricular hemorrhage. Characteristics of the 45 infants are listed in Table 3. Thus, apnea and intraventricular hemorrhage were subsequently applied as additional risk factors of TR-ROP to improve the sensitivity of the DIGIROP-Birth model. Infants reported to be at risk below 0.84% by the DIGIROP-Birth model were reclassified as high-risk for TR-ROP. The sensitivity of the modified DIGIROP-Birth model that included these additional risk factors increased to 89/93 (95.7%), and the NPV was 263/267 (98.5%).

**Table 2:** Performances of the DIGIROP-Birth model for TR-ROP in the study cohort (*n* = 442)

Cutoff	0.0084 probability	0.0076 probability	0.0083 probability	0.0089 probability	0.0200 probability
Sensitivity	48/93 (51.6%)	49/93 (52.7%)	48/93 (51.6%)	46/93 (49.5%)	32/93 (34.4%)
Specificity	263/349 (75.4%)	257/349 (73.6%)	263/349 (75.4%)	263/349 (75.4%)	311/349 (89.1%)
PPV	48/134 (35.8%)	49/141 (34.8%)	48/134 (35.8%)	46/132 (34.8%)	32/70 (45.7%)
NPV	263/308 (85.4%)	257/301 (85.4%)	263/308 (85.4%)	263/310 (84.8%)	311/372 (83.6%)

*TR-ROP* Treatment-requiring retinopathy of prematurity, *PPV* Positive predictive value, *NPV* Negative predictive value

**Table 3:** Characteristics of the 45 TR-ROP infants reported to be at risk below 0.84%

Infant no./sex	GA, wk. + d	BW, g	Apnea	Intraventricular hemorrhage	Risk prediction
1/Female	29 + 5	1160	Y	N	0.0007
2/Male	29 + 5	1400	Y	Y	0.0022
3/Female	27 + 6	1100	N	Y	0.0060
4/Female	30 + 1	1420	N	Y	0.0003
5/Male	30 + 1	1300	N	N	0.0010
6/Female	30 + 3	1150	Y	N	0.0004
7/Male	28 + 5	1250	Y	N	0.0059
8/Female	30 + 3	1210	N	N	0.0003
9/Male	29 + 5	1180	N	Y	0.0030
10/Female	30 + 6	900	N	N	0.0004
11/Female	29 + 6	1600	Y	Y	0.0004
12/Male	30 + 1	1350	N	N	0.0018
13/Female	30 + 3	1390	Y	N	0.0003
14/Male	29 + 6	1030	Y	Y	0.0035
15/Male	29 + 1	1300	N	Y	0.0039
16/Female	28 + 3	1300	N	Y	0.0024
17/Female	30 + 0	1100	Y	N	0.0006
18/Female	30 + 5	1100	N	Y	0.0003
19/Male	30 + 2	1640	N	Y	0.0011
20/Female	28 + 4	1080	N	Y	0.0027
21/Female	29 + 2	1400	N	Y	0.0008
22/Female	29 + 5	1600	Y	Y	0.0004
23/Female	28 + 1	1000	Y	N	0.0050
24/Female	28 + 0	1000	Y	N	0.0059
25/Male	30 + 0	1630	N	Y	0.0013
26/Female	28 + 1	990	Y	Y	0.0051
27/Female	29 + 0	1400	Y	Y	0.0011
28/Male	30 + 3	1800	Y	N	0.0008
29/Male	30 + 3	1600	N	Y	0.0010
30/Female	29 + 0	1200	N	Y	0.0014
31/Female	29 + 0	1100	Y	N	0.0017
32/Female	30 + 0	1350	Y	N	0.0004
33/Male	29 + 3	1160	N	Y	0.0039
34/Female	29 + 1	1200	Y	N	0.0012
35/Female	29 + 4	1200	N	Y	0.0008
36/Female	30 + 2	990	N	Y	0.0005
37/Male	28 + 6	1580	Y	Y	0.0037
38/Female	27 + 5	1025	Y	N	0.0078
39/Female	29 + 3	1200	Y	Y	0.0009
40/Female	28 + 3	1220	Y	N	0.0026
41/Male	28 + 6	1130	N	Y	0.0063
42/Female	28 + 0	1030	Y	N	0.0055
43/Female	27 + 6	1160	N	Y	0.0056
44/Male	29 + 3	1410	Y	Y	0.0027
45/Female	28 + 5	1070	Y	N	0.0024

*BW* Birth weight, *GA* Gestational age, *N* No, *Y* Yes

The performance of the DIGIROP-Birth model for identifying TR-ROP infants was also evaluated by gestational age and birth weight (Tables 4, 5, 6 and 7). In the 100 infants with a gestational age < 28 weeks, when applying a cutoff value of 0.0084, the sensitivity increased from 48/93 (51.6%) to 36/39 (92.3%), and the NPV was 4/7 (57.1%). In the 342 infants with a gestational age ≥ 28 weeks, the sensitivity was 12/54 (22.2%), and NPV was 259/301 (86.0%). In the 60 infants with a birth weight < 1000 g, a sensitivity of 20/23 (87.0%) and an NPV of 6/9 (66.7%) were obtained. Finally, among the 382 infants with a birth weight ≥ 1000 g, the sensitivity was 28/70 (40.0%), and the NPV was 257/299 (86.0%).

**Table 4:** Performances of the DIGIROP-Birth model for TR-ROP in the infants < 28 weeks (*n* = 100)

Cutoff	0.0084 probability	0.0076 probability	0.0083 probability	0.0091 probability	0.0200 probability
Sensitivity	36/39 (92.3%)	37/39 (94.9%)	36/39 (92.3%)	36/39 (92.3%)	31/39 (79.5%)
Specificity	4/61 (6.6%)	2/61 (3.3%)	4/61 (6.6%)	4/61 (6.6%)	23/61 (37.7%)
PPV	36/93 (38.7%)	37/96 (38.5%)	36/93 (38.7%)	36/93 (38.7%)	31/69 (44.9%)
NPV	4/7 (57.1%)	2/4 (50.0%)	4/7 (57.1%)	4/7 (57.1%)	23/31 (74.2%)

*TR-ROP* Treatment-requiring retinopathy of prematurity, *PPV* Positive predictive value, *NPV* Negative predictive value

**Table 5:** Performances of the DIGIROP-Birth model for TR-ROP in the infants ≥28 weeks (*n* = 342)

Cutoff	0.0084 probability	0.0076 probability	0.0083 probability	0.0091 probability	0.0200 probability
Sensitivity	12/54 (22.2%)	12/54 (22.2%)	12/54 (22.2%)	10/54 (18.5%)	1/54 (0.3%)
Specificity	259/288 (89.9%)	255/288 (88.5%)	259/288 (89.9%)	262/288 (91.0%)	288/288 (100.0%)
PPV	12/41 (29.3%)	12/45 (26.7%)	12/41 (29.3%)	10/36 (27.8%)	1/1 (100.0%)
NPV	259/301 (86.0%)	255/297 (85.9%)	259/301 (86.0%)	262/306 (85.6%)	288/341 (84.5%)

*TR-ROP* Treatment-requiring retinopathy of prematurity, *PPV* Positive predictive value, *NPV* Negative predictive value

**Table 6:** Performances of the DIGIROP-Birth model for TR-ROP in the infants < 1000 g (*n* = 60)

Cutoff	0.0084 probability	0.0076 probability	0.0083 probability	0.0091 probability	0.0200 probability
Sensitivity	20/23 (87.0%)	20/23 (87.0%)	20/23 (87.0%)	19/23 (82.6%)	18/23 (78.3%)
Specificity	6/37 (16.2%)	6/37 (16.2%)	6/37 (16.2%)	6/37 (16.2%)	12/37 (32.4%)
PPV	20/51 (39.2%)	20/51 (39.2%)	20/51 (39.2%)	19/50 (38.0%)	18/43 (41.9%)
NPV	6/9 (66.7%)	6/9 (66.7%)	6/9 (66.7%)	6/10 (60.0%)	12/17 (70.6%)

*TR-ROP* Treatment-requiring retinopathy of prematurity, *PPV* Positive predictive value, *NPV* Negative predictive value

**Table 7:** Performances of the DIGIROP-Birth model for TR-ROP in the infants ≥1000 g (*n* = 382)

Cutoff	0.0084 probability	0.0076 probability	0.0083 probability	0.0091 probability	0.0200 probability
Sensitivity	28/70 (40.0%)	29/70 (41.4%)	28/70 (40.0%)	27/70 (38.6%)	14/70 (20.0%)
Specificity	257/312 (82.4%)	251/312 (80.4%)	257/312 (82.4%)	260/312 (83.3%)	299/312 (95.8%)
PPV	28/83 (33.7%)	29/90 (32.2%)	28/83 (33.7%)	27/79 (34.2%)	14/27 (51.9%)
NPV	257/299 (86.0%)	251/292 (86.0%)	257/299 (86.0%)	260/303 (85.8%)	299/355 (84.2%)

*TR-ROP* Treatment-requiring retinopathy of prematurity, *PPV* Positive predictive value, *NPV* Negative predictive value

## Discussion

Visual loss from ROP may be prevented by early diagnosis and timely treatment, which emphasizes the importance of ROP screening in routine clinical practice [1]. Recently, Pivodic et al. developed an individual risk prediction model, DIGIROP-Birth, using only birth characteristics to describe a continuous hazard function for identifying TR-ROP [23]. This easy-to-use prediction model was built using Swedish National Patient Registry data, and validated in US and European cohorts, yielding satisfactory results. The present study validated the DIGIROP-Birth model in Chinese preterm infants, and found that the model had less satisfactory performance than previously reported (AUC = 0.634 in this study vs. AUC = 0.85 in the study by Pivodic et al.) [23].

Several reasons could account for the discrepancy. First, there were only 83 (5.4%) Asians among the 1535 infants that comprised the US validation group in Pivodic et al.’s study [23], while our study cohort consisted of 442 Chinese infants. Asian infants appear to be at higher risk of developing TR-ROP than white infants due to differences in ethnic ancestry and underlying genetic predisposition [20, 21]. Second, compared with the DIGIROP-Birth model’s training cohort, our cohort of Chinese infants had an older mean gestational age (28.8 weeks vs. 28.1 weeks) and lower mean birth weight (1119 g vs. 1237 g). This might be explained by the fact that in less-developed countries, severe ROP occurs in more mature and larger infants [3]. Third, the quality of neonatal care is one of the most critical factors for ROP development and progression [28]. Besides a lack of neonatologists and nurses with neonatal care expertise, neonatal units are in short supply of enough equipment for continuous monitoring of preterm infants on supplemental oxygen [29–31]. The timing and duration of supplemental oxygen, oxygen concentration, and prolonged mechanical ventilation are among the most crucial risk factors for TR-ROP [32]. Consequently, preterm infants born in less-developed countries or regions are more likely to be exposed to postnatal risk factors for TR-ROP that are better controlled in industrialized countries [29, 31]. Similar characteristics of ROP and the corresponding clinical settings have been reported in other developing countries in Asia and Latin America [29–31]. Finally, the DIGIROP-Birth model did not consider postnatal risk factors for ROP, which could also account for its decreased predictive performance in our Chinese cohort. Modification of the DIGIROP-Birth model through the incorporation of postnatal risk factors might improve its applicability in less-developed countries.

Several studies have shown that complications of prematurity, such as bronchopulmonary dysplasia, apnea, intraventricular hemorrhage, and sepsis, are associated with the development of ROP [32–35]. Gestational age, birth weight, male sex, apnea, and intraventricular hemorrhage were found to be independent risk factors for TR-ROP in our cohort of Chinese infants with gestational ages of 24 to 30 weeks. Therefore, apnea and intraventricular hemorrhage were included as additional risk factors in our modified DIGIROP-Birth model. Our principal goal was to determine the sensitivity of the DIGIROP-Birth model. That is, its ability to rule out TR-ROP and determine the number of ROP screening examinations that could have been safely spared by using this model. With the cutoff value of 0.0084, the sensitivity of the DIGIROP-Birth model improved from 51.6 to 95.7%, with an NPV of 98.5%. Previous studies also revealed that apnea of prematurity and intraventricular hemorrhage were independently associated with a higher risk of ROP [34–36]. Infants with apnea are more likely to require oxygen therapy, which could induce ROP development due to immature antioxidant systems. Oxygen-related factors play a crucial role in TR-ROP, including the duration of supplemental oxygen, oxygen concentration, and prolonged mechanical ventilation [32]. Although several large randomized-controlled studies have compared different oxygen saturation target ranges, the ideal range that could reduce ROP occurrence without increasing preterm infants’ mortality remains controversial [37–40]. Intraventricular hemorrhage occurs in 25–30% of preterm infants with birth weights < 1500 g, often causing neurodevelopmental impairment [41]. Early control of intracranial pressure secondary to intraventricular hemorrhage may prevent TR-ROP development in infants with a combined diagnosis of ROP and intraventricular hemorrhage. This is because the progression of ROP may associate with reduced ocular circulation secondary to high intracranial pressure [42]. Thus, apnea and intraventricular hemorrhage, two important premature birth complications, could greatly improve the predictive ability of the DIGIROP-Birth model for TR-ROP in Chinese preterm infants. However, no significance was observed in other important risk factors included in our study, such as respiratory distress syndrome, anaemia, or invasive mechanical ventilation. This could be explained by the small sample size.

The sensitivity of the DIGROP-Birth model in infants with a gestational age < 28 weeks or a birth weight < 1000 g was satisfactory. This suggests that the DIGROP-Birth model may also be valuable as an auxiliary tool for ROP screening in extremely preterm infants and infants with extremely low birth weight. In infants with a gestational age ≥ 28 weeks or birth weight ≥ 1000 g, the DIGIROP-Birth model was less effective, but could be modified with postnatal risk factors. Thus, the DIGIROP-Birth model still has the potential to decrease the frequency of ROP examinations in less-developed countries.

There was another factor impeding the generalization of the DIGROP-Birth model to Chinese preterm infants. The DIGROP-Birth model was developed in Swedish infants with gestational ages less than 31 weeks, thus this model could not be applied to infants with gestational ages of 31 weeks or more [23], resulting in 38.2% of infants eligible for ROP screening to be excluded from our validation study. Nevertheless, TR-ROP could occurs in these Chinese infants with higher gestational ages [4], prediction models are needed to identify these subset of infants at high risk. The performance of the DIGIROP-Birth model is worthy of validation in infants with gestational ages beyond 30 weeks, and might be also modified with postnatal risk factors.

This study has several limitations. First, this validation study was conducted retrospectively. Despite the retrospective nature, however, the clinical data included in our analyses are routinely recorded in the neonatal intensive care units and could be collected reliably. Second, the single-center study had a relatively small sample size compared with other validation studies of ROP prediction models. Future multicenter prospective studies with large cohorts will enhance our findings.

## Conclusions

This study validated the DIGIROP-Birth model in a cohort of Chinese preterm infants. Although the predictive performance of the model was lower than in developed countries, modification of the model with postnatal risk factors showed promise for improving its efficacy for TR-ROP. The DIGIROP-Birth model was also effective in infants with a younger gestational age or with extremely low birth weight. We believe that more studies in less-developed countries and other races will improve the efficacy and generalizability of the DIGIROP-Birth model, and subsequent adjustment of the easy-to-use model will help reduce the frequency of ROP screening examinations.

## Supplementary Information

**Additional file 1: Supplementary Table 1.** Univariate and multivariate analysis of risk factors for TR-ROP.

## Data Availability

The datasets generated during and analyzed during the current study are not publicly available due to the reason that the data also forms part of an ongoing study but are available from the corresponding author on reasonable request.

## Abbreviations

AUC: Area under the receiver-operating characteristic curve
CI: Confidence interval
NPV: Negative predictive value
OR: Odds ratio
PPV: Positive predictive value
ROC: Receiver-operating characteristic
ROP: Retinopathy of prematurity
TR-ROP: Treatment-requiring retinopathy of prematurity
