# Supplementary material for: Validation of the DIGIROP-birth model in a Chinese cohort
Source: BMC Ophthalmol. 2021 May 27;21:236. doi: 10.1186/s12886-021-01952-0 (PMC8161896; doi:10.1186/s12886-021-01952-0)
Supplement: Supplementary file 1 — Additional file 1: Supplementary Table 1. Univariate and multivariate analysis of risk factors for TR-ROP. [file 12886_2021_1952_MOESM1_ESM.docx]

**Title:** **Validation of the DIGIROP-Birth Model in a Chinese Cohort**

**Authors:** Sizhe Chen^#^, Rong Wu^#^, He Chen, Wenbei Ma, Shaolin Du, Chao Li, Xiaohe Lu^*^, Songfu Feng^*^.

^#^ These authors contributed equally to this work.

^*^ Xiaohe Lu and Songfu Feng should be co-corresponding author.

**Supplementary Table 1.** Univariate and multivariate analysis of risk factors for TR-ROP.

| Variables | Univariate analysis | | | Multivariate analysis | | |
| --- | --- | --- | --- | --- | --- | --- |
|  | *P* | OR | 95% CI | *P* | Adjusted OR | 95% CI |
| **Maternal factors** | | | | | | |
| Maternal age, years |  |  |  |  |  |  |
| 25–30 | Reference | | | Reference | | |
| <25 | 0.761 | 1.111 | 0.563–2.194 | Excluded from multivariate analyses | | |
| 30–35 | 0.540 | 1.196 | 0.674–2.124 | Excluded from multivariate analyses | | |
| >35 | 0.513 | 1.258 | 0.663–2.499 | Excluded from multivariate analyses | | |
| Caesarean delivery | 0.185 | 0.718 | 0.441–1.171 | Excluded from multivariate analyses | | |
| Multiple birth | 0.144 | 0.636 | 0.347–1.166 | Excluded from multivariate analyses | | |
| In vitro fertilization | 0.679 | 0.857 | 0.413–1.778 | Excluded from multivariate analyses | | |
| Gestational hypertension | 0.179 | 1.708 | 0.782–3.727 | Excluded from multivariate analyses | | |
| Gestational diabetes | 0.877 | 1.051 | 0.563–1.961 | Excluded from multivariate analyses | | |
| Reproductive tract infections during pregnancy | 0.277 | 0.583 | 0.220–1.543 | Excluded from multivariate analyses | | |
| Antenatal steroids use | 0.082 | 0.577 | 0.310–1.073 | 0.091 | 0.564 | 0.291–1.095 |
| **Neonatal factors** | | | | | | |
| Gestational age, weeks | <0.001 | 0.614 | 0.512–0.737 | 0.034 | 0.758 | 0.587–0.979 |
| Birth weight, g | <0.001 | 0.223 | 0.101–0.547 | 0.016 | 0.261 | 0.107–0.635 |
| Male | 0.047 | 1.604 | 1.006–2.557 | 0.030 | 1.767 | 1.058–2.952 |
| Apnea | 0.043 | 1.641 | 1.017–2.648 | 0.020 | 2.013 | 1.305–3.156 |
| Respiratory distress syndrome | 0.287 | 1.362 | 0.771–2.407 | Excluded from multivariate analyses | | |
| Bronchopulmonary dysplasia | 0.077 | 1.543 | 0.955–2.494 | 0.664 | 1.400 | 0.852–2.300 |
| Sepsis | 0.844 | 0.951 | 0.575–1.572 | Excluded from multivariate analyses | | |
| Necrotizing enterocolitis | 0.203 | 1.541 | 0.793–2.996 | Excluded from multivariate analyses | | |
| Intraventricular hemorrhage | 0.035 | 1.641 | 1.035–2.602 | 0.009 | 3.617 | 1.365–8.521 |
| Patent ductus arteriosus | 0.796 | 1.064 | 0.665–1.703 | Excluded from multivariate analyses | | |
| Anaemia | 0.804 | 1.079 | 0.591–1.970 | Excluded from multivariate analyses | | |
| Hyperbilirubinemia | 0.419 | 0.826 | 0.519–1.313 | Excluded from multivariate analyses | | |
| **Neonatal interventions** | | | | | | |
| Invasive mechanical ventilation | 0.885 | 1.071 | 0.424–2.701 | Excluded from multivariate analyses | | |
| Blood transfusions | 0.082 | 2.343 | 0.899–6.108 | 0.080 | 2.445 | 0.899–6.649 |

CI, confidence interval; OR, odds ratio; TR-ROP, treatment-requiring retinopathy of prematurity.
